# Mental Health Services for Serious Mental Illness: Scoping Review of Randomised Controlled Trials

**DOI:** 10.1002/nop2.70100

**Published:** 2025-01-04

**Authors:** Pablo Roson Rodriguez, Xiao Chen, Marcelo Arancibia, Eva Madrid, Farhad Shokraneh, Clive E. Adams, Juan Víctor Ariel Franco

**Affiliations:** ^1^ Research Department Cochrane Argentina Associate Centre—Instituto Universitario Hospital Italiano de Buenos Aires Buenos Aires Argentina; ^2^ Minerva Schools at Keck Graduate Institute San Francisco California USA; ^3^ Interdisciplinary Centre for Health Studies (CIESAL) Universidad de Valparaíso, Cochrane Chile Associate Centre, Viña del Mar Valparaiso Chile; ^4^ Cochrane Schizophrenia, Institute of Mental Health, Division of Psychiatry and Applied Psychology, School of Medicine University of Nottingham Nottingham UK; ^5^ Institute of General Practice Medical Faculty of the Heinrich‐Heine‐University Düsseldorf Düsseldorf Germany

**Keywords:** health services delivery, public health, randomised trial, schizophrenia, service evaluation

## Abstract

**Aims:**

This review aims to classify the evidence from randomised controlled trials (RCTs) on mental health services (MHS) for people with serious mental illness (SMI) available in the Cochrane Schizophrenia Group's (CSzG) specialised register.

**Design:**

Scoping review.

**Methods:**

We retrieved and screened RCTs of service‐level interventions considering non‐pharmacological approaches for mental healthcare of the CSzG register. We classified and collected the main characteristics of the RCTs using a customised data extraction and charting form based on DESDE‐LTS classification.

**Results:**

We included 233 out of 262 total trial registries. Most of the studies were conducted in China, 136 (58%), 57 (24%) North America and 26 (11%) Europe. We classified the studies as ambulatory assistance 80 (34%), day services/out‐patient care 38 (16%), residential services 44 (19%), accessibility to care 19 (8%), information/assessment 39 (17%), self‐help and voluntary help 10 (4%), e‐health 52 (22%), and discharge services 17 (7%).

**Conclusions:**

We found a large number of trials that investigated the effects of mental health services for people with SMI. Trials classification was difficult due to the poor report of the characteristics of these complex interventions. This database can be used to plan and prioritise systematic reviews according to the needs of stakeholders.

**Relevance Statement:**

The study is of interest to mental health nursing because it studies the different services in which nurses play a fundamental role with implications in the nursing practice, education, research or leadership and management.

## 1 Background

In this review, serious mental illness (SMI) is defined as a mental, behavioural or emotional disorder resulting in serious functional impairment, which substantially interferes with or limits one or more major life activities (National Institute of Mental Health (NIMH) 2023). SMI includes mental disorders such as schizophrenia, affective disorders (including depressive, mania and bipolar disorder) and other psychotic diseases. This group comprises emotional, cognitive or behavioural disturbances, often resulting in permanent functional disability in daily life activities (Reilly et al. 2013; Zumstein and Riese 2020).

Since the 1970s, European countries began a trend towards deinstitutionalization that led to the closure of inpatient centres and the creation of a wide variety of community services to care for patients (Kunitoh 2013). These new community services involve a high cost for health systems (Reilly et al. 2013; Zumstein and Riese 2020; Gustavsson et al. 2011; Ride et al. 2020). Mental health services are micro‐level systems of organisation of mental health care with administrative structure and own personnel that are concerned with the evaluation, diagnosis, monitoring and treatment of patients with mental illness (Salvador‐Carulla et al. 2011; Queensland Health 2014). Most of them are classified as ‘complex interventions’, distinguished by presenting a great variety of procedures and stakeholders (Castelpietra et al. 2017). Due to the difficulty in their classification, the European Union has created different tools for this purpose, such as the Description and Standardised Evaluation of Services and Directories in Europe for Long‐Term Care (DESDE‐LTC). This tool uses a system of trees or diagrams that allows the categorisation of services and the level of use thereof by users in the selected area (DESDE‐LTC n.d.). Efforts to classify mental health services respond to the need to avoid ambiguities in their categorisation, which is extremely important for researchers, clinicians and policymakers in order to assess the evidence for these interventions, allocate resources and assess later on their effectiveness (Castelpietra et al. 2017).

The Cochrane Schizophrenia Group (CSzG) is part of the international collaborative organisation Cochrane, an independent not‐for‐profit consortium dedicated to providing accurate and updated information about the effects of healthcare, free of conflict of interests. The CSzG is concerned with evaluating the prevention, treatment and rehabilitation of people with SMI. The CSzG has published several maps of RCTs using the group's comprehensive specialised trial register. The topics included: treatment‐resistant schizophrenia, pharmacological treatments, psychotherapies and traditional Chinese medicine interventions for people with schizophrenia (Deng and Adams 2017; Sinclair 2014; Roberts et al. 2021; Shokraneh and Adams 2021). These maps may help design and prioritise relevant systematic reviews, especially when synthesising evidence from complex interventions in which their categorisation might pose challenges for the review question (Schuller‐Martínez et al. 2021). Nonetheless, previous scoping reviews on this topic have been restricted to a reduced group of interventions such as home‐ and community‐based interventions for recovery (Bitter et al. 2020; Bruce, Van Citters, and Bartels 2005), technology (Lal et al. 2023), occupational therapy (Conn et al. 2019), self‐care promotion (Strunz et al. 2022); or were limited to a geography (Chimara, Van Niekerk, and van Biljon 2022; Elman et al. 2019). Therefore, no broad scoping review mapped the wide variety of mental health services for people with severe mental illness.

## 2 Aims

To produce a scoping review of the available evidence from randomised controlled trials through the classification of the mental health services for mental health services interventions for SMI. This review serves as a go‐to resource for evidence on these interventions to inform evidence‐based policy and plan evidence synthesis.

## 3 Methods

We use the scoping review design to describe the body of evidence available in mental health services for SMI. This study design (‘Big Picture Review’) allows to map the breadth of evidence in a particular field, clarifying concepts, definitions and, in our case, classifications (Campbell et al. 2023). This study was carried out following the Global Evidence Mapping Initiative (GEM) methodology and the authors' other mapping studies' methods to categorise the evidence, but as this is a scoping review, we did not provide a visual representation of the data (Sinclair 2014; Madera Anaya et al. 2019). All methods were specified in advance and documented in a protocol. The protocol will not be registered in PROSPERO because this platform does not register scoping review; however, we uploaded the protocol in the Open Science Framework (Data S1).

### 3.1 Eligibility Criteria

#### 3.1.1 Inclusion Criteria

- Study design: Randomised controlled trials (RCTs) retrieved from the CSzG register. We included randomised controlled trials because they are the gold standard study design to assess the effects of interventions. Considering that many of these are complex interventions, this also encompasses cluster randomised controlled trials, which are a common design to assess the effectiveness of health services.
- Type of population: People with SMI, including mental disorders such as schizophrenia, affective disorders (including depressive, mania and bipolar disorder) and other psychotic diseases. This group comprises emotional, cognitive or behavioural disturbances, often resulting in permanent functional disability in daily life activities (Reilly et al. 2013; Zumstein and Riese 2020).
- Type of interventions: RCTs with a broad definition of service‐level interventions, considering non‐pharmacological approaches for mental healthcare care in patients with SMI, such as arrangements for accessibility and delivery of care (for instance, early referral, transitional care and the use of technology), residential services and education and promotion of self‐help and peer‐support. This correlates with other definitions of services used in international classifications (see below) (Salvador‐Carulla et al. 2011).

#### 3.1.2 Exclusion Criteria

- Trials that have been classified in previously published maps with a different scope: treatment‐resistant schizophrenia, pharmacological treatments, psychotherapies and traditional Chinese medicine.

### 3.2 Search Strategy

CSzG has developed and maintained the largest database of randomised clinical trial (RCT) reports and studies of people with SMI. This register contains 27,861 reports for 19,964 coded studies (May 22, 2019) (Shokraneh and Adams 2020). The register is maintained using a relational database, MeerKat 1.6, which stores the references as studies (without DESDE‐LTC classification) and is updated daily (Shokraneh and Adams 2017). The group also uses the Cochrane Register of Studies to deliver some records to the Cochrane Central Register of Controlled Trials (CENTRAL). Because of copyrighted materials and lack of technological support in the Cochrane Register of Studies for some languages, such as the Chinese language, CENTRAL covers only about 20% of references from the register (Cochrane Schizophrenia n.d.).

The CSzG register was searched by the information specialist (FS) in February 2019 (Register of Trials n.d.). This register is compiled by systematic searches of 70 different biomedical databases, including AMED, BIOSIS, CENTRAL, CINAHL, ClinicalTrials.gov, ProQuest's Dissertations and Theses, Embase, ISRCTN, LILACS, MEDLINE, PsycINFO, PubMed, WHO ICTRP and is supplemented with hand searching of relevant journals and numerous conference proceedings. This strategy attempts to reduce the risk of publication bias. A detailed account of the group's search strategy is available. The search terms used were: nurse, interface, liaison, crisis, team, home, case, day hospital, community, rehabilitation, support, and program—service (Register of Trials n.d.; Search Strategy n.d.). The information specialist marked the interventions that are likely to be relevant to service level among 2800 interventions with the tag ‘{SER}’ in the database and then retried all the trials linked to this tag. The marking was a sensitive effort to cover even slightly relevant interventions or the interventions that cannot be decided to be service level or not in the search.

### 3.3 Selection of the Studies

**FIGURE 1:**
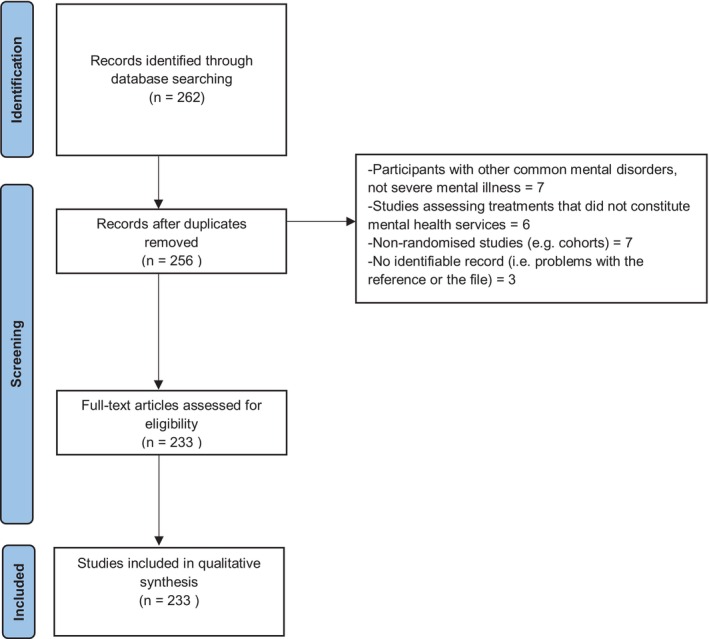
Study flow diagram.

We handled all the retrieved titles and abstracts with the reference manager software Rayyan (Ouzzani et al. 2016). After removing duplicates, three reviewers (M.A., P.R. and X.C.) independently screened all titles/abstracts to exclude irrelevant studies. Then, full articles were obtained for a final decision. Details of reasons for exclusion of any study considered relevant were clearly stated. We present the study flow diagram (see Figure 1).

### 3.4 Data Extraction

We collected data using a customised data extraction form, which was tested to ensure that the process would be performed consistently among reviewers. Data were collected on the following levels:

- General characteristics of the study: authors, year of publication, type of RCT (parallel, cross‐over, individual or cluster randomisation), objective, number of participants included and the main conclusion.
- Characteristics of the leading research question: we identified the main research question of each study based on the main aim stated by the authors, the eligibility criteria and its conclusions. The research questions were drawn using the PICO framework, which specifies the four key components of a well‐defined question on interventions: population, intervention, comparison and outcomes. We then extracted details on the population characteristics, the intervention and the comparator.
- Characteristics of other research questions from the study: we considered secondary research questions if the article described all the elements of the PICO question and a conclusion about the direction of the effect. We extracted the same information described above for the main research question.

Three authors working in pairs (P.R., M.A., X.C.) independently performed all processes of selection of studies and data extraction. If there were any disagreements, they were resolved by consensus and, when necessary, an additional reviewer (J.V.A.F.) participated in the discussion until an agreement was reached. If required, we contacted RCT authors for clarification or to obtain missing information. Multiple reports of single trials were grouped to avoid double counting (a single RCT may appear in numerous publications and, if not corrected for, might introduce spurious precision by being counted over and over again).

### 3.5 Charting and Presentation

We classified all the trials in the selected categories taking into account the PICO questions of the included RCTs, creating a detailed database that is uploaded into the Open Science Framework (https://osf.io/btyn6/). We then summarised the study characteristics per category (age, diagnosis, etc.) in a table. The main findings and characteristics of the included RCTs were analysed in each of the DESDE‐LTC categories. The intent of this study is not to evaluate the interventions themselves if not to evaluate the main characteristics of the available RCT evidence in each category to guide evidence synthesis and uptake. Finally, we present a summary of the review according to the PAGER framework, considering Patterns, Advances, Gaps, Evidence for Practice and Research recommendations (Bradbury‐Jones et al. 2022; Bradbury‐Jones and Aveyard 2021).

### 3.6 DESDE‐LTC Classification

This classification is an instrument for the standardised description and classification of long‐term care (LTC) services in Europe. DESDE‐LTC classifies services considering them a ‘micro‐level of organisation and administrative unit encompassing a group of organised structures and professionals that provide care (Salvador‐Carulla et al. 2011). This classification follows an approach developed by the European Psychiatric Assessment Team (EPCAT) and PSICOST group (Romero‐López‐Alberca et al. 2019). This tool intends, through categorisation, to gather information on inputs and processes at the level of individual services and the level of health or social areas. The services it aims to classify are those intended for adults with physical or mental disabilities. Our review aims to classify trials related to mental health services, so the classification provided by DESDE‐LTC was the basis for categorising the trial interventions. Through an iterative process, the lead author classified each trial into categories with the consultation of the other authors involved in the data extraction (M.A. and X.C.) and the supervision of the senior author (J.V.A.F.). We created two additional categories to group interventions that were not adequately represented in the DESDE‐LTC classification, and we deemed it relevant to report separately (discharge and e‐Health).

As this scoping review is not intended to assess the effectiveness of each intervention we did not critically appraise the included studies nor performed qualitative or quantitative synthesis (Khalil and Tricco 2022; Schuller‐Martínez et al. 2021). The results of this study were reported according to the PRISMA‐ScR extension for scoping studies (Tricco et al. 2018).

## 4 Results

We retrieved 262 studies from the register; seven reports were merged with other studies and 23 were excluded. We included 233 studies in our review (Figure 1). Most of the RCTs used a parallel design (*n* = 222; 95%), followed by cluster (*n* = 11; 5%). Most of the studies were conducted in China (*n* = 136; 58%), North America (*n* = 57; 24%) and Europe (*n* = 26; 11%), or more than one high‐income country (*n* = 2; 1%), with only 2.6% from low and middle‐income countries (five from Iran and one from Indonesia). The rest of the studies were conducted in Australia (*n* = 4; 2%) and Korea (*n* = 1; 0.4%). Regarding the setting, 78% were in outpatient departments, 13% in‐hospital patients and 9% in both outpatient and inpatient settings.

The mean number of participants was 174 (interquartile range (IQR) 25–75: 80–200). Most of the studies included adult participants without distinction of age or sex (97%). Although all the studies recruited participants with SMI, some studies used specific subpopulations (24%): 8% evaluated the first psychotic episode, followed by 4% of the studies in participants who were homeless or at risk of becoming. Other subpopulations such as ‘substance use problems’ (2%) or veterans (2%) were also evaluated in a much smaller proportion.

See Table 1 for the general details on the populations, settings and interventions.

**TABLE 1:** Characteristics of RCT and interventions.

**Study population and setting**	
Sample size (mean and IQ range)	174 (80–200)
Special population (e.g., military, homeless, only women, adolescents)—*n* (%)	56/233 (24%) First psychotic episode: 18 (8%) Homeless: 10 (4%) Women: 5 (2%) Patient with addiction: 4 (2%) Veterans: 4 (2%) Elderly adults: 3 (1%) Others: 11 (5%)
Setting—*n* (%)	21 (9%) inpatient/outpatient 31 (13%) inpatient 181 (78%) outpatient
**Study interventions**	
Provider—*n* (%)	83 (36%) Nursing staff 109 (47%) Mental health team Others: miscellaneous (Volunteers, etc.)
Classification in one of the DESDE‐LTC categories—*n* (%)	Ambulatory assistance: 80 (34%) e‐health: 52 (22%) Residencial services: 44 (19%) Information/assessment: 40 (17%) Day services/Outpatient care: 38 (16%) Accessibility to care: 19 (8%) Discharge service: 17 (7%) Self‐help and voluntary help: 10 (4%)
Number of classifications per study—*n* (%)	One classification 177 (76%) Two classifications 46 (20%) Three classifications 8 (3%) Four classifications 2 (1%)
Follow‐up—*n* (%)	< 6 months: 50 (22%) 6 to 12 months: 70 (30%) 12 to 24 months: 54 (23%) > 24 months: 12 (5%) Unclear: 47 studies (20%)

## 5 Classification of Mental Health Services

### 5.1 Ambulatory Assistance

The DESDE‐LTC includes those interventions in which there is contact between staff and users associated with clinical or social difficulties, and the interventions are not provided as part of residential or day services. Many of these interventions include a component of community participation. We found significant heterogeneity in the nature and description of the interventions. Furthermore, they were poorly reported, which hindered our efforts to draw sub‐classifications. We found 80 (34%) studies, of which 51% evaluated interventions in acute participants, 46% in non‐acute participants and the remainder in both (3%). We found 59 (74%) studies in which the intervention was mobile. The other 19 studies (24%) described interventions developed in the community or hospital centres. Mobile interventions were delivered either by a nurse staff or a community mental health team in the participant's houses, mainly offering support and follow‐up or directly providing rehabilitation in different modalities (family education, case management or assertive community treatment). The non‐mobile interventions were primarily delivered by a mental health team (47%) to provide follow‐up, skills training and medication adherence assessment.

### 5.2 Day Services

The DESDE‐LTC day services category contains interventions in which the facilities are available in regular opening hours to several users at the same time. Generally, they also provide treatment for problems related to long‐term care needs through structured activities or social support. We included 38 studies in this category, of which 26 (68%) were directed to chronic or subacute participants, including skills training and day hospital. The other 12 (32%) targeted acute participants and included vocational orientation and social/educational programmes.

### 5.3 Residential Services

The DESDE‐LTC residential services category includes those which provide beds to carry outpatient care, excluding those for the homeless. This review includes housing intervention for homeless people with SMI because it is an important field of study within community mental health research. It is important to clarify that not all interventions carried out in a hospital setting, mostly with hospitalised people, were classified as residential interventions, but only the interventions specifically assessed residential interventions. Within the housing category, we found the ‘Housing First model intervention’, which is one of the most widely used boarding models and is a standardised model providing a housing solution based on the harm reduction approach, the empowerment of users in their treatment and the fact that the house is separated from treatment. This intervention model includes housing with a rental subsidy and follow‐up using support services through assertive community treatment teams or case managers.

We included 44 (19%) studies in this category: 20 studies (47%) were classified as housing intervention and 24 (53%) in the residential intervention; 17 of these were with acute patients while the remaining seven were with non‐acute patients; 13 (76%) of the acute care residential services were carried out in a hospital setting while the remaining study corresponded to a community crisis house. Five of the residential intervention studies for non‐acute patients were carried out in community settings.

- Within the *non‐acute patients* (seven studies) in community settings, we had the characteristic of being unlocked residences with the follow‐up monitored by community mental health teams. Two of these are declared as halfway houses, and another two are residential crisis programmes and centres for independent living. Two of these included peer support as an important part of the intervention.
- Of the 17 interventions for *acute patients*, four of them were interventions in which the usual patterns of hospitalisation were modified (pre‐programmed hospitalisations, double free day, discharge on parole and mixed rooms), while the remaining were adherence to treatment or rehabilitation (*n* = 4) and improvement of nursing care intervention (*n* = 9).
- Housing interventions are those whose primary purpose is to provide a housing solution to homeless users or users at risk of being homeless. We included 20 trials in this category, seven (35%) focused on Housing First. The other 13 (65%) studies focused on the housing solution along with a variety of community follow‐up interventions.

### 5.4 Accessibility to Care

This category includes studies whose primary intervention facilitated access to different services they require without necessarily providing them. Within this category, the most widely used intervention model was ‘case management’, characterised by planning and coordination of the care required to meet the needs of patients. Generally, this model of care is carried out by a nurse or a social worker. These interventions are designed at the community level, where they supervise and articulate with the different services and levels the set of benefits that the patient requires. We included 19 (8%) studies in this category, of which 13 (68%) were described as case management or similar, and 6 (32%) performed other interventions focused on coordination and accessibility.

### 5.5 Information and Assessment

Those interventions whose main objective was to provide information, training, education or evaluate and subsequent orientation of users without necessarily monitoring them were classified here. Interventions focused on assessment included those in which professionals assessed and subsequently guided the users. We included 40 (17%) RCT in this category:

- Information (*n* = 15; 38%): 13 trials were conducted in an outpatient setting; Most of them were family and patient education interventions, including cognitive training, financial coaching intervention, dental care education, telephone psycho‐education, among others. The only RCT conducted in a hospital setting focused on mental health education and the improvement of the quality of service.
- Assessment (*n* = 25; 62%): 15 trials were in an inpatient setting; 10 of these had the focus on health assessment, two on the nursing assistance and three were on quality control interventions; 10 trials were in an outpatient setting; five of these focusing on health assessment and the other three on early detection of the condition (including early interventions for people with psychosis), quality of life and therapeutic adherence assessments.

### 5.6 Self‐Help and Voluntary Help

We included within these interventions those that provide support, self‐help and accompaniment to users by non‐professional personnel, users or former users. The DESDE‐LTC classification also takes the criterion that the staff is unpaid; we did not take this criterion in this work. We found 10 studies in this category (4.3%). These studies included: support by volunteers in psychiatric wards (*n* = 2), patients and former patients in community settings (*n* = 5) and peers linked to discharge planning (*n* = 3).

## 6 Other Categories

### 6.1 Discharge Services

We defined discharge interventions as those aimed at facilitating patient discharge. Discharge interventions have the characteristic of focusing on the transitional care of patients who are discharged. Mainly the interventions include pre‐discharge planning as a post‐discharge follow‐up of patients in the community. Seventeen (7%) studies were classified in this category. The RCT evaluated two types of interventions: Eleven studies evaluated supporting patients after discharge, and six studies evaluated interventions supporting transitional discharge, including discharge planning and community support.

### 6.2 E‐Health

This category includes all the care services that depend on modern information and communication technologies (ICT). This may include interventions in which services within other overlapping categories include ICT or those primarily delivered through ICT. We included 52 (22%) trials in this category. These studies assessed interventions that used ICT with different purposes: 56% to enhance follow‐up, 15% to provide education, 10% to send reminders to improve adherence and 6% to perform assessments. Some interventions had more than one purpose: 4% follow‐up plus education and 10% reminders plus follow‐up.

### 6.3 PAGER Framework

A summary of the review in terms of Patterns, Advances, Gaps, Evidence for Practice and Research recommendations (PAGER) can be found in Table 2.

**TABLE 2:** Summary of the review according to the PAGER framework.

Patterns	Advances	Gaps	Evidence	Research recommendations
Population	The recovered studies mostly evaluated participants with SMI, finding 24% of the special population: 8% first psychotic episode, 4% homeless, 2% women, 2% addicts and 1% older adults.	We found a lack of evidence related to treatment‐resistant patients.	Evidence from Cochrane reviews indicate that intensive case management, crisis intervention, day centres, peer support, educational interventions and training kills programmes may reduce hospitalisations, relapse, functioning and improve mental health.	Research related to treatment‐resistant participants is needed. Future research should also incorporate greater detail of the participants' diagnoses.
Setting	Mostly out‐patient services were evaluated (78%).	There is a lack of evidence on community services for chronic patients such as halfway houses.		Future research should explore community interventions in chronic patients who are underrepresented.
Classification	A wide variety of outpatient (34%) and e‐health (22%) services were studied.	Lack of evidence about hospital discharge services.		It is necessary to develop research related to hospital discharge services and mutual support services.

## 7 Discussion

Our research included 233 RCTs, mostly with parallel design. The majority of the studies analysed a single intervention provided by the mental health teams or nurses, with a follow‐up of 6–12 months. Interventions were categorised according to the DESDE‐ LCT classification. Through the description and classification of RCTs in mental health services for SMI made in our map, we try to offer an overview of the existing evidence in the field of mental health services, so that it is useful both for researchers, clinicians and policymakers as a basic input to have an approximation to the state of the art (or to the main characteristics) of the different branches of interest, as to recognise gaps in evidence in mental health services. We also found that research in this area was concentrated in China, North America and Europe, which highlights the need for further research in different settings.

### 7.1 Related Synthesised Evidence

For ambulatory assistance care, most of the studies evaluated mobile interventions (74%) delivered at the participants' house and related to different dimensions of rehabilitation, including some components of community participation. However, their characteristics and descriptions were very heterogeneous. These interventions make up an interesting area for future evidence synthesis with the challenge of achieving a convenient way to categorise them. A systematic review discussed the impact of the lack of precision of the classifications and nomenclature of the interventions on misleading research findings (Catty et al. 2007). Several Cochrane reviews cover these topics in SMI: intensive case management (Dieterich et al. 2017), crisis intervention (Murphy et al. 2015) and educational interventions (Sin et al. 2015; Xia, Merinder, and Belgamwar 2011). Although the evidence of the systematic reviews is of low to moderate certainty, the results indicate that these approaches effectively improve compliance and functioning and reduce days of hospitalisation.

Almost all‐day services focused on chronic patients, defined as those whose illness has been present for at least 6 months (O'Halloran, Miller, and Britt 2004), developed interventions such as skills training for long‐term care. A systematic review on this topic found that day hospitals compared to admission for acute psychiatric disorders results in little to no differences in unemployment, quality of life or satisfaction (Marshall et al. 2011). Another Cochrane review found limited evidence of the effectiveness of programmes promoting different types of life skills (interpersonal, stress management, financial, etc.) (Tungpunkom, Maayan, and Soares‐Weiser 2012).

Concerning housing and residential services, most of the included studies were classified as housing interventions, among which the most frequent were those targeted to acutely ill patients and those under the Housing First model. For this model, we found a systematic review that included studies on people with mental disorders, substance abuse and dual disorders; this review found that the intervention effectively reduced homelessness and improved housing stability (Gulcur et al. 2003). We also found other systematic reviews of housing programmes, which demonstrated moderate certainty of the evidence that housing interventions could be more effective than residential care in housing status, social integration, health status, quality of life, satisfaction and cost (Newman 2001; Richter and Hoffmann 2017; Rog et al. 2014). Mixed‐methods research of supported housing for people with SMI highlights the difficulty of keeping patients motivated and the importance of offering an individualised approach so that they remain active both inside and outside of supported housing facilities (Tjörnstrand et al. 2020).

Case management was the main intervention we identified under accessibility to care, focusing on integrating services for rehabilitation and follow‐up, combining home visits, coordination of care between healthcare providers, among other components (Salvador‐Carulla et al. 2011). A Cochrane review on intensive case management for SMI found low certainty evidence that this intervention effectively reduces hospitalisations, increases retention in care and improves functioning (Dieterich et al. 2017).

For studies analysing services focused on assessment, most of them assessed the participants' risk behaviours, compliance with care and early detection. We found several reviews that examined instruments for assessing risk behaviours (Ayhan and Üstün 2021; Janse van Rensburg and van der Wath 2020; Large et al. 2016), but none assessed the effects of implementing them in people with SMI. A Cochrane review found very low certainty evidence that training to recognise the early signs of recurrence in schizophrenia may positively affect relapse and re‐hospitalised rates (Morriss et al. 2013). For information, most studies focused on skill training through e‐health. A Cochrane review found very low certainty of evidence that psychoeducational interventions through ICT may improve patients' mental health, with little to no difference in other outcomes (Välimäki et al. 2012).

E‐health interventions are a growing field of research due to the emerging new technologies and end‐users technology acceptance (Lawes‐Wickwar, McBain, and Mulligan 2018). Telemedicine can be used for monitoring chronic health conditions and providing treatment, education and advice for self‐management (Flodgren et al. 2015). Low certainty evidence indicates that telephone and remote medication monitoring may be effective for people with SMI, while the other related interventions (delivery of patient education using computers) showed little to no difference compared with the standard care. Cochrane reviews found very low certainty of the evidence for effects of telemedicine for other health conditions, except those targeting patients with heart problems where they are probably effective in reducing cardiovascular risks (Bittner et al. 2015; Flodgren et al. 2015; Flumignan et al. 2019; Gentry et al. 2013; Inglis et al. 2015; Kew and Cates 2016; Khan et al. 2015; Laver et al. 2013; McLean et al. 2011; Tan and Lai 2012).

The category self‐help and voluntary help included interventions related to peer support. We found several systematic reviews on this field with a wide range of purposes and modalities (Campos et al. 2014; Fortuna et al. 2020; Repper and Carter 2011; Ridout and Campbell 2018; Walker and Bryant 2013; White et al. 2020). Most of them were delivered in community settings, in groups by former patients, patients or caregivers, together with e‐health support, among others. A Cochrane review found very low certainty evidence on its effectiveness on relapse, hospitalisation and mortality (Chien et al. 2019).

For interventions targeting the discharge process, some included pre‐discharge planning and post‐discharge follow‐up. Three systematic reviews found that these may effectively reduce re‐hospitalisation and improve adherence (Petkari et al. 2020; Steffen et al. 2009; Tyler, Wright, and Waring 2019). Our team recently published a systematic review on transitional discharge interventions and found no clear evidence for or against their implementation (Roson Rodriguez et al. 2024).

Finally, it is important to highlight the central role that mental health nurses (MHNs) play in mental health services. We found that 36% of RCTs have MHN as their primary provider, not counting the important role they play within mental health teams. It is remarkable how, despite the evident importance of MHNs in all mental health service settings, they are still not recognised in treatment programmes with the importance they deserve (Hurley et al. 2022).

### 7.2 Limitations

The limitations of the study were related to the difficulties in the classification of RCTs, since the tool DESDE‐LTC was originally conceived to classify health services and not RCTs, which led us to create new categories to allow a better representation of the diversity of included interventions. Finally, we were unable to find studies in low‐ and middle‐income countries, therefore there might be uncertainties regarding the generalizability or applicability of these interventions in other contexts. Moreover, this highlights the need for high‐quality randomised controlled trials in these settings. No classification system has been identified to cover all variants of health services for people with severe mental illnesses. We identified these emerging categories through multiple iterations that we assessed as useful for policymakers and other stakeholders. We acknowledge that these categories could be challenged. Still, considering that we provide all the detailed information of the included studies in these categories, this allows for a transparent assessment of their suitability.

One of the strengths of the review was the search on the exhaustive trial register, which is compiled through systematic searches in 70 different biomedical databases together with manual searches of grey literature, which attempts to mitigate the risk of publication bias. Furthermore, using the DESDE‐LTC registry as a basis for classifying the RCTs can link the evidence to a familiar classification for decision‐makers. Finally, to ensure the fidelity of these findings, all extractions were done in duplicate and classifications were done as an iterative process, incorporating a native Chinese speaker as an author for the assessment of studies in Chinese studies.

For future research in the field of mental health services, it is important to improve the quality of the reports through the use of validated tools such as ‘Criteria for Reporting the Development and Evaluation of Complex Interventions in healthcare’ (CReDECI 2) or ‘Template for Intervention Description and Replication’ (TIDieR) (Hoffmann et al. 2014; Möhler, Köpke, and Meyer 2015).

## 8 Conclusions

We found a large number of trials that investigated the effects of mental health services for people with SMI. Trials classification was difficult due to the poor report of the characteristics of these complex interventions. This database can be used to plan and prioritise systematic reviews according to the needs of stakeholders.

## Author Contributions

**Pablo Roson Rodriguez:** conceptualization, formal analysis, investigation, methodology, project administration, validation, writing original draft preparation, writing – review and editing. **Xiao Chen:** investigation, validation, writing original draft preparation, writing – review and editing. **Marcelo Arancibia:** investigation, validation, writing original draft preparation, writing. **Eva Madrid:** supervision, writing – review and editing. **Farhad Shokraneh:** conceptualization, data curation, writing original draft preparation. **Clive E. Adams:** conceptualization, supervision. **Juan Víctor Ariel Franco:** conceptualization, formal analysis, investigation, methodology, project administration, supervision, writing original draft preparation, writing – review and editing.

## Conflicts of Interest

The authors declare no conflicts of interest.

## Supporting information


Data S1.

## Data Availability

The complete database is available at https://osf.io/btyn6/.
