# Supplementary material for: Mental Health Services for Serious Mental Illness: Scoping Review of Randomised Controlled Trials
Source: Nurs Open. 2025 Jan 4;12(1):e70100. doi: 10.1002/nop2.70100 (PMC11702393; doi:10.1002/nop2.70100)
Supplement: Supplementary file 1 — Data S1. [file NOP2-12-e70100-s001.docx]

**Mental health services for serious mental illness: A map of randomised controlled trials**

**(Protocol)**

**Background**

In this review, serious mental illness (SMI) is defined as a mental, behavioural, or emotional disorder resulting in serious functional impairment, which substantially interferes with or limits one or more major life activities [(*National Institute of Mental Health (NIMH)*, n.d.)](https://paperpile.com/c/uHIX9D/bNQZ). SMI includes various mental disorders such as schizophrenia, affective disorders (including depressive, mania and bipolar disorder) and other psychotic diseases. This group comprises emotional, cognitive or behavioural disturbances, often resulting in permanent functional disability in daily life activities [(Reilly et al., 2013; Zumstein & Riese, 2020)](https://paperpile.com/c/uHIX9D/G4uyA+dCJq5). For this reason, a wide variety of devices and interventions are used for their treatment which means a high cost for the health systems [(Gustavsson et al., 2011; Ride et al., 2020)](https://paperpile.com/c/uHIX9D/iAaja+eEiy4). Since the 1970s, European countries began a tendency towards deinstitutionalization, i.e. treatment of SMI carried out in community devices. A wide variety of strategies have been created [(Kunitoh, 2013)](https://paperpile.com/c/uHIX9D/CFWwx). Most of them are classified as ‘complex interventions’, distinguished by presenting a great variety of procedures and stakeholders [(Castelpietra et al., 2017)](https://paperpile.com/c/uHIX9D/kEFB). Due to the difficulty in their classification, the European Union has created different tools for this purpose, such as the Description and Standardized Evaluation of Services and Directories in Europe for Long-Term Care (DESDE-LTC). This tool uses a system of trees or diagrams that allows the categorisation of services and the level of use thereof by users in the selected area [(*eDesde*, n.d.)](https://paperpile.com/c/uHIX9D/BSu1p). Efforts to classify mental health services respond to the need to avoid ambiguities in their categorization, which is extremely important for researchers, clinicians and policymakers in order to assess the evidence for these interventions, allocate resources, and assess later on their effectiveness (Castelpietra et al., 2017).

The Cochrane Schizophrenia Group (CSzG) is part of the international collaborative organisation Cochrane, an independent not-for-profit consortium dedicated to providing accurate and updated information about the effects of healthcare, free of conflict of interests. The CSzG is concerned with evaluating the prevention, treatment, and rehabilitation of people with SMI. It has developed and maintained the largest database of randomised clinical trial (RCT) reports and studies of people with SMI. This register contains 27,861 reports for 19,964 coded studies (May 22, 2019) [(Shokraneh & Adams, 2020)](https://paperpile.com/c/uHIX9D/aImYu). The register is maintained using a relational database, MeerKat 1.6, which stores the references as studies (without DESDE-LTC classification), and is updated daily [(Shokraneh & Adams, 2017)](https://paperpile.com/c/uHIX9D/Kx2Fm). The group also uses the Cochrane Register of Studies to deliver some records to the Cochrane Central Register of Controlled Trials (CENTRAL). Because of copyrighted materials and lack of technological support in the Cochrane Register of Studies for some languages, such as the Chinese language, CENTRAL covers only about 20% of references from the register [(*Cochrane Schizophrenia*, n.d.)](https://paperpile.com/c/uHIX9D/43GfC).

The CSzG has published several maps of RCTs using the group's comprehensive specialised trial register. The topics included: treatment-resistant schizophrenia, pharmacological treatments, psychotherapies and traditional Chinese medicine interventions for people with schizophrenia [(Deng & Adams, 2017; Diarmid Sinclair, 2014; Roberts et al., 2021; Shokraneh & Adams, 2021)](https://paperpile.com/c/uHIX9D/P8rAF+4heCL+bTgsY+ZWPKk). These maps may help design and prioritise relevant systematic reviews, especially when synthesising evidence from complex interventions in which their categorisation might pose challenges for the review question[(Schuller-Martínez et al., 2021)](https://paperpile.com/c/uHIX9D/8UDJa).

**Aims**
To produce a map of the available randomised evidence through the classification of the mental health services for mental health services interventions for SMI. This map serves as a go-to resource for evidence on these interventions to inform evidence-based policy and plan evidence synthesis.

**Methods**
This mapping study will carry out following the Global Evidence Mapping Initiative (GEM) methodology and the authors’ other mapping studies’ methods [(Diarmid Sinclair, 2014; Madera Anaya et al., 2019)](https://paperpile.com/c/uHIX9D/IOcvF+ZWPKk). The protocol will not be registered in PROSPERO because this platform does not register evidence maps.

*Eligibility criteria*

We will assess RCTs from the CSzG register. After excluding the classes of trials that have been classified in previously published maps (on treatment-resistant schizophrenia, pharmacological treatments, psychotherapies and traditional Chinese medicine), the information specialist will mark the interventions that are likely to be relevant to service level among 2,800 interventions as '{SER} and then will retry all the trials linked to this tag. The marking is a sensitive effort to cover even slightly relevant interventions or the interventions that cannot be decided to be service level or not in the search.

We will include RCTs with a broad definition of service-level interventions, considering non-pharmacological approaches for mental healthcare such as arrangements for accessibility and delivery of care (for instance, early referral, transitional care and the use of technology), residential services, and education and promotion of self-help and peer-support. This correlates with other definitions of services used in international classifications (see below) [(Carulla et al., 2011)](https://paperpile.com/c/uHIX9D/TXLo1).

*Search strategy*

The CSzG register will be searched by the information specialist (FS) in February 2019 [(*Register of Trials*, n.d.)](https://paperpile.com/c/uHIX9D/Cn5mX). This register is compiled by systematic searches of 70 different biomedical databases, including AMED, BIOSIS, CENTRAL, CINAHL, ClinicalTrials.Gov, ProQuest’s Dissertations and Theses, Embase, ISRCTN, LILACS, MEDLINE, PsycINFO, PubMed, WHO ICTRP, and is supplemented with hand searching of relevant journals and numerous conference proceedings. This strategy attempts to reduce the risk of publication bias. A detailed account of the group’s search strategy will be available.

*Selection of the studies*
We handled all the retrieved titles and abstracts with the reference manager software Rayyan [(Ouzzani et al., 2016)](https://paperpile.com/c/uHIX9D/vDTJ3). After removing duplicates, three reviewers (MA, PR and XC) independently will screen all titles/abstracts to exclude irrelevant studies. Then, full articles will be obtained for a final decision. Details of reasons for exclusion of any study considered relevant will be clearly stated. We will present the study flow diagram.

*Data extraction*
We will collect data using a customised data extraction form, which will be tested to ensure that the process would be performed consistently among reviewers. Data will be collected on the following levels:

- General characteristics of the study: authors, year of publication, type of RCT (parallel, cross-over, individual or cluster randomisation), objective, number of participants included and the main conclusion.
- Characteristics of the leading research question: We will identify the main research question of each study based on the main aim stated by the authors, the eligibility criteria, and its conclusions. The research questions will be drawn using the PICO framework, which specifies the four key components of a well-defined question on interventions: population, intervention, comparison, and outcomes. Then we will extract details on the population characteristics, the intervention and the comparator.
- Characteristics of other research questions from the study: We will consider secondary research questions if the article described all the elements of the PICO question and a conclusion about the direction of the effect. We will extract the same information described above for the main research question.

Three authors working in pairs (PR, XC, MA) independently will perform all processes of selection of studies and data extraction. If there are any disagreements, they will be resolved by consensus, and, when necessary, an additional reviewer (JVAF) will participate in the discussion until an agreement will be reached. If required, we will contact RCT authors for clarification or obtain missing information. Multiple reports of single trials will be grouped to avoid double counting (a single RCT may appear in numerous publications and, if not corrected for, might introduce spurious precision by being counted over and over again).
*Evidence mapping presentation*

We will present the evidence mapping through a table describing the characteristics of the included RCTs and with the characteristics of all PICOs identified. Trials will be presented according to categories of similar populations and interventions.

*DESDE-LTC classification*

This classification is an instrument for the standardised description and classification of Long-Term Care (LTC) services in Europe. DESDE-LTC classifies services considering them a ‘micro-level of organisation and administrative unit encompassing a group of organized structures and professionals that provide care[(Carulla et al., 2011)](https://paperpile.com/c/uHIX9D/TXLo1). This classification follows an approach developed by the European Psychiatric Assessment Team (EPCAT) and PSICOST group[(Romero-López-Alberca et al., 2019)](https://paperpile.com/c/uHIX9D/ivos4). This tool intends, through categorisation, to gather information on inputs and processes at the level of individual services and the level of health or social areas. The services it aims to classify are those intended for adults with physical or mental disabilities. Our map will aim to classify trials related to mental health services, so the classification provided by DESDE-LTC will be the basis for categorising the trial interventions. Through an iterative process, the lead author will classify each trial into categories with the consultation of the other authors involved in the data extraction (MA and XC) and the supervision of the senior author (JVAF).

As this map does not intend to assess the effectiveness of each intervention we will not critically appraise the included studies nor perform qualitative or quantitative synthesis [(Schuller-Martínez et al., 2021)](https://paperpile.com/c/uHIX9D/8UDJa). The results of this study will be reported according to the PRISMA-ScR extension for scoping studies[(Tricco et al., 2018)](https://paperpile.com/c/uHIX9D/zoQGE).

**References**

[Carulla, L. S., Dimitrov, H., Weber, G., McDaid, D., Venner, B., Šprah, L., Romero, C., Ruiz, M., Tibaldi, G., & Johnson, S. (2011). *DESDE-LTC: Evaluation and Classification of Services for Long Term Care in Europe*.](http://paperpile.com/b/uHIX9D/TXLo1)

[Castelpietra, G., Salvador-Carulla, L., Almborg, A.-H., Fernandez, A., & Madden, R. (2017). Working draft: Classifications of interventions in mental health care. An expert review. *The European Journal of Psychiatry*, *31*(4), 127–144.](http://paperpile.com/b/uHIX9D/kEFB)

[*Cochrane schizophrenia*. (n.d.). Retrieved May 19, 2021, from](http://paperpile.com/b/uHIX9D/43GfC) <https://schizophrenia.cochrane.org/register-trials>

[Deng, H., & Adams, C. E. (2017). Traditional Chinese medicine for schizophrenia: A survey of randomized trials. *Asia-Pacific Psychiatry: Official Journal of the Pacific Rim College of Psychiatrists*, *9*(1). https://doi.org/](http://paperpile.com/b/uHIX9D/P8rAF)[10.1111/appy.12265](http://dx.doi.org/10.1111/appy.12265)

[Diarmid Sinclair, C. E. A. (2014). Treatment resistant schizophrenia: a comprehensive survey of randomised controlled trials. *BMC Psychiatry*, *14*. https://doi.org/](http://paperpile.com/b/uHIX9D/ZWPKk)[10.1186/s12888-014-0253-4](http://dx.doi.org/10.1186/s12888-014-0253-4)

[*eDesde*. (n.d.). Retrieved May 7, 2021, from](http://paperpile.com/b/uHIX9D/BSu1p) <http://www.edesdeproject.eu/>

[Gustavsson, A., Svensson, M., Jacobi, F., Allgulander, C., Alonso, J., Beghi, E., Dodel, R., Ekman, M., Faravelli, C., Fratiglioni, L., Gannon, B., Jones, D. H., Jennum, P., Jordanova, A., Jönsson, L., Karampampa, K., Knapp, M., Kobelt, G., Kurth, T., … CDBE2010Study Group. (2011). Cost of disorders of the brain in Europe 2010. *European Neuropsychopharmacology: The Journal of the European College of Neuropsychopharmacology*, *21*(10), 718–779.](http://paperpile.com/b/uHIX9D/eEiy4)

[Kunitoh, N. (2013). From hospital to the community: the influence of deinstitutionalization on discharged long-stay psychiatric patients. *Psychiatry and Clinical Neurosciences*, *67*(6), 384–396.](http://paperpile.com/b/uHIX9D/CFWwx)

[Madera Anaya, M., Franco, J. V. A., Ballesteros, M., Solà, I., Urrútia Cuchí, G., & Bonfill Cosp, X. (2019). Evidence mapping and quality assessment of systematic reviews on therapeutic interventions for oral cancer. *Cancer Management and Research*, *11*, 117–130.](http://paperpile.com/b/uHIX9D/IOcvF)

[*National Institute of Mental Health (NIMH)*. (n.d.). Retrieved February 21, 2022, from](http://paperpile.com/b/uHIX9D/bNQZ) <https://www.nimh.nih.gov/health/statistics/mental-illness#:~:text=Serious%20mental%20illness%20(SMI)%20is,or%20more%20major%20life%20activities.>

[Ouzzani, M., Hammady, H., Fedorowicz, Z., & Elmagarmid, A. (2016). Rayyan-a web and mobile app for systematic reviews. *Systematic Reviews*, *5*(1), 210.](http://paperpile.com/b/uHIX9D/vDTJ3)

[*Register of trials*. (n.d.). Retrieved May 21, 2021, from](http://paperpile.com/b/uHIX9D/Cn5mX) <https://schizophrenia.cochrane.org/register-trials>

[Reilly, S., Planner, C., Gask, L., Hann, M., Knowles, S., Druss, B., & Lester, H. (2013). Collaborative care approaches for people with severe mental illness. *Cochrane Database of Systematic Reviews* , *11*, CD009531.](http://paperpile.com/b/uHIX9D/G4uyA)

[Ride, J., Kasteridis, P., Gutacker, N., Aragon Aragon, M. J., & Jacobs, R. (2020). Healthcare Costs for People with Serious Mental Illness in England: An Analysis of Costs Across Primary Care, Hospital Care, and Specialist Mental Healthcare. *Applied Health Economics and Health Policy*, *18*(2), 177–188.](http://paperpile.com/b/uHIX9D/iAaja)

[Roberts, M. T., Shokraneh, F., Sun, Y., Groom, M., & Adams, C. E. (2021). Classification of psychotherapy interventions for people with schizophrenia: development of the Nottingham Classification of Psychotherapies. *Evidence-Based Mental Health*, *24*(2), 62–69.](http://paperpile.com/b/uHIX9D/bTgsY)

[Romero-López-Alberca, C., Gutiérrez-Colosía, M. R., Salinas-Pérez, J. A., Almeda, N., Furst, M., Johnson, S., & Salvador-Carulla, L. (2019). Standardised description of health and social care: A systematic review of use of the ESMS/DESDE (European Service Mapping Schedule/Description and Evaluation of Services and DirectoriEs). *European Psychiatry: The Journal of the Association of European Psychiatrists*, *61*, 97–110.](http://paperpile.com/b/uHIX9D/ivos4)

[Schuller-Martínez, B., Meza, N., Pérez-Bracchiglione, J., Franco, J. V. A., Loezar, C., & Madrid, E. (2021). Graficando el cuerpo de la evidencia: lo esencial para comprender el enfoque de los mapas de brecha de evidencia. *Medwave*, *21*(03). https://doi.org/](http://paperpile.com/b/uHIX9D/8UDJa)[10.5867/medwave.2021.03.8164](http://dx.doi.org/10.5867/medwave.2021.03.8164)

[Shokraneh, F., & Adams, C. E. (2017). Study-based registers of randomized controlled trials: Starting a systematic review with data extraction or meta-analysis. *BioImpacts : BI*, *7*(4), 209–217.](http://paperpile.com/b/uHIX9D/Kx2Fm)

[Shokraneh, F., & Adams, C. E. (2020). Cochrane Schizophrenia Group’s Study-Based Register of randomized controlled trials: Development and content analysis. *Schizophrenia Bulletin Open*, *1*(1). https://doi.org/](http://paperpile.com/b/uHIX9D/aImYu)[10.1093/schizbullopen/sgaa061](http://dx.doi.org/10.1093/schizbullopen/sgaa061)

[Shokraneh, F., & Adams, C. E. (2021). Classification of all pharmacological interventions tested in trials relevant to people with schizophrenia: A study-based analysis. *Health Information and Libraries Journal*. https://doi.org/](http://paperpile.com/b/uHIX9D/4heCL)[10.1111/hir.12366](http://dx.doi.org/10.1111/hir.12366)

[Tricco, A. C., Lillie, E., Zarin, W., O’Brien, K. K., Colquhoun, H., Levac, D., Moher, D., Peters, M. D. J., Horsley, T., Weeks, L., Hempel, S., Akl, E. A., Chang, C., McGowan, J., Stewart, L., Hartling, L., Aldcroft, A., Wilson, M. G., Garritty, C., … Straus, S. E. (2018). PRISMA Extension for Scoping Reviews (PRISMA-ScR): Checklist and Explanation. *Annals of Internal Medicine*, *169*(7), 467–473.](http://paperpile.com/b/uHIX9D/zoQGE)

[Zumstein, N., & Riese, F. (2020). Defining Severe and Persistent Mental Illness—A Pragmatic Utility Concept Analysis. In *Frontiers in Psychiatry* (Vol. 11). https://doi.org/](http://paperpile.com/b/uHIX9D/dCJq5)[10.3389/fpsyt.2020.00648](http://dx.doi.org/10.3389/fpsyt.2020.00648)
